# Measuring the quality of Patients’ goals and action plans: development and validation of a novel tool

**DOI:** 10.1186/1472-6947-12-152

**Published:** 2012-12-27

**Authors:** Cayla R Teal, Paul Haidet, Ajay S Balasubramanyam, Elisa Rodriguez, Aanand D Naik

**Affiliations:** 1 Houston VA HSR&D Center of Excellence, Michael E. DeBakey VA Medical Center, Houston, TX, USA; 2 Department of Medicine, Section of Health Services Research, Baylor College of Medicine, MEDVAMC HSR&D Center of Excellence, 2002 Holcombe Blvd. (152), Houston, TX, 77030, USA; 3 Department of Medicine, Section of General Medicine, and the Office of Undergraduate Medical Education, Baylor College of Medicine, Houston, TX, USA; 4 The Office of Medical Education and the Departments of Medicine and Humanities, The Pennsylvania State University College of Medicine, Hershey, PA, USA

**Keywords:** Goal-setting, Diabetes, Self-management, Goals, Action plans, Measurement

## Abstract

**Background:**

The purpose of this study is to develop and test reliability, validity, and utility of the Goal-Setting Evaluation Tool for Diabetes (GET-D). The effectiveness of diabetes self-management is predicated on goal-setting and action planning strategies. Evaluation of self-management interventions is hampered by the absence of tools to assess quality of goals and action plans. To address this gap, we developed the GET-D, a criteria-based, observer rating scale that measures the quality of patients’ diabetes goals and action plans.

**Methods:**

We conducted 3-stage development of GET-D, including identification of criteria for observer ratings of goals and action plans, rater training and pilot testing; and then performed psychometric testing of the GET-D.

**Results:**

Trained raters could effectively rate the quality of patient-generated goals and action plans using the GET-D. Ratings performed by trained evaluators demonstrated good raw agreement (94.4%) and inter-rater reliability (Kappa = 0.66). Scores on the GET-D correlated well with measures theoretically associated with goal-setting, including patient activation (r=.252, P<.05), diabetes specific self-efficacy (r=.376, P<.001) and inverse relationship with depression (r= −.376, P<.01). Significant between group differences (P<.01) in GET-D scores between goal-setting intervention (mean = 7.33, standard deviation = 4.4) and education groups (mean = 4.93, standard deviation = 3.9) confirmed construct validity of the GET-D.

**Conclusions:**

The GET-D can reliably and validly rate the quality of goals and action plans. It holds promise as a measure of intervention fidelity for clinical interventions that promote diabetes self-management behaviors to improve clinical outcomes.

**Trial registration:**

Clinicaltrials.gov Identifier: NCT00481286

## Background

The Chronic Care model provides a framework for improving diabetes care that espouses ongoing interactions between a prepared, proactive team of clinicians and an informed activated patient [1]. Key outputs of this “activated” interaction are the setting of collaborative, patient-centered, management goals (goal-setting) and feedback regarding structured activities patients do on a daily basis to reach their self-management goals (action plans) [2]. Goal setting and action planning are not new concepts in diabetes care [3] and are routinely discussed as essential and largely inseparable components. Developing goals and action plans has long been promoted as a principal element of effective diabetes self-management programs [4,5]. However, empirical evidence to support the efficacy of goal-setting in diabetes care has been equivocal at best [5,6]. This mixed performance possibly stems from the inconsistency of how goal-setting interventions are conducted and the lack of validated tools to measure the quality of the goals and action plans that comprise such interventions [7].

Traditional assessments of diabetes care, including aspects of collaborative goal-setting, have focused primarily on the structure and process of chronic care management. In many studies, assessments of goal setting included only patient reports about the presence or absence of a discussion about or the development of collaborative goals [8]. Comparatively little attention has been given to the *content* and *quality* of the goals and action plans themselves measured against theoretically-grounded criteria. Since the quality of patient goals and action plans is related to their achievement [9,10], definitive evaluation of the relationship between goal-setting and diabetes outcomes (goal-attainment) cannot be accomplished without a valid measure of the quality of goals and action plans that result from collaborative goal-setting.

To address this critical gap, we developed a criteria-based, observer rating scale that measures the quality of patients’ diabetes goals and action plans (Goal Evaluation Tool for diabetes GET-D) based on goal-setting theory. In this article, we describe the development process and assessment of the GET-D’s reliability and validity.

## Methods

The GET-D was developed to assess written goals and action plans that result from interventions or encounters designed to facilitate goal setting. Development included: a) identification of relevant criteria and scoring weights for use in rating patients’ goal and action plans, with associated rater instructions and training; b) pilot testing and revision of the GET-D criteria, scoring weights, rater instructions and rater training; and c) psychometric testing of the GET-D using existing data drawn from a clinical trial. This study was approved by the Baylor College of Medicine Institutional Review Board.

### GET-D criteria identification and scoring

To identify criteria that define high-quality goals, and establish content validity, we began with a review of key behavioral change theories related to goal setting and action planning, Locke & Latham’s Goal Setting Theory [11] and Schwarzer’s Health Action Process Approach [12]. Goal Setting Theory identifies two specific attributes of goals – specificity and difficulty. The Health Action Process Approach identifies at least three attributes of action planning – when, where, and how a goal will be achieved. We then undertook a review of articles specifically related to goal-setting in the context of diabetes and health self-management, strictly for the purpose of identifying how these attributes were operationalized and to lend more detail to the instrument being developed. To identify these articles, we conducted a series of key word searches (“goal setting”, “action plan” combined with “patient”) in Pub Med and Psych Info, each limited to the previous fifteen years’ articles. These searches revealed 683 unique articles. The abstracts were then reviewed to identify articles discussing conceptual descriptions of or educational programs teaching adult patients effective goal-setting or action planning. This resulted in 114 (16.7%) articles, which were then reviewed.

This review, with discussion of the research team, resulted in the creation of ten criteria for the instrument, three for goal setting and seven for action planning. Effective goals for diabetes care were operationalized as having three criteria [3,7,13,14], each related to the “specificity” attribute of goals described by Locke and Latham [11]. First, the goal must be related to a self-management task that could potentially affect diabetes outcomes. Second, the goal must have measurable specificity, that is, specify an outcome that could be observed or measured to track one’s success. Third, the presence of a specific deadline or target end date for meeting the goal was essential. After much discussion about expected challenges in having raters score difficulty of goals (which depended on information about the goal-setter that would not be typically available to those assessing goal quality), our team elected not to include the “difficulty” attribute of goals described in Goal Setting Theory.

Effective action plans were operationalized as having seven additional criteria [10,15-18]. First, previous data collection by our research team suggested that the first criterion should assess if the action plan directly related to the goal that had been set. The second through sixth criteria referenced five specific elements, including a) explicit actions that will be carried out (related to the “how” attribute of action planning described by Schwarzer) [12] b) the frequency or schedule on which the actions will be carried out (related to Schwarzer’s “when” attribute of action planning), c) the location in which the action is carried out (related to the “where” attribute), d) the intensity or duration of the actions (related to the “how” attribute), and e) how the activity will be monitored or tracked (a criteria suggested by data demonstrating the effectiveness of monitoring in goal achievement) [11]. The seventh criterion was whether the action plan was feasible for the patient to carry out, which was suggested as a pilot item by our research team in response to observing numerous patients’ plans.

**Figure 1:**
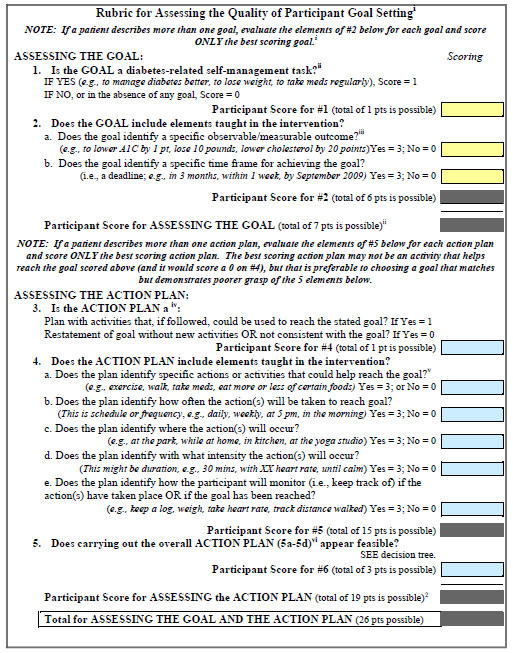
Final Goal-Setting Evaluation Tool (GET-D).

Once these initial criteria were identified, we considered mechanisms for rating or scoring each criterion about patients’ written goals and action plans. As shown in Figure 1, in the final GET-D, criteria were rated according to their presence or absence in the goal or action plan. We posited that if most of these criteria were present, a rater should be able to judge the feasibility of the action plan, such as in item 5 of Figure 1. Feasibility ratings were expected to answer the question of “Can the action plan be reached if put into practice just as written?” We developed a one-page guide of detailed scoring instructions (see Additional file 1: Appendix), as well as a practical rater training session.

### Source of patient goals and action plans: the *empowering patient in collaborative care* (EPIC) study

The *Empowering Patient in Collaborative Care* (EPIC) [19] is a randomized comparative effectiveness study involving primary care patients with type 2 diabetes recruited from a diabetes registry of a large regional health system (additional details of study participants were provided in the prior publication). EPIC participants were randomized to two groups using block randomization, each receiving distinct approaches to group diabetes self-management, described previously [19]. After the active intervention period, all EPIC participants completed a comprehensive assessment including providing written answers to two open-ended questions related to diabetes goals and action plans they set during the intervention: 1) “Being as specific and detailed as possible please describe your diabetes goals” and 2) “Being as specific and detailed as possible, what actions will you take to reach your diabetes goal?” We utilized these written goals and action plans, which were selected and written by the participants independent of assistance from professionals, for the psychometric evaluation of the GET-D.

### Pilot testing and modifications to GET-D, rater instructions and training

The objectives for the pilot test were 1) to assess inter-rater reliability of the GET-D among multiple raters using patients’ hand-written goals and action plans, and 2) to obtain raters’ feedback on the rater instructions and training sessions and on the usefulness of the GET-D. Seven raters were trained in a group session and then asked to independently rate five patient-generated, hand-written goals and their associated action plans. All raters were compared to each other rater.

To carry out the first objective, we examined raw agreement and inter-rater reliability (using a multi-rater, multi-category Kappa) [20] for each criterion, averaging these findings. We found consistency across the eleven GET-D criteria as evidenced by strong average raw agreement among raters (80.0%) and good average inter-rater reliability (Kappa = 0.67). Nine of the ten criteria (i.e., those that were dichotomous) had ratings that clustered around the same score (0, 1, or 3). However, feasibility ratings showed greater discrepancies between the raters. To promote inter-rater reliability, we developed a decision tree to guide raters while assessing action planning feasibility; these were included in the rater instructions. When all modifications to the GET-D were made, the Goal-Setting subtotal included a possible 7 points and the Action Planning subtotal included a possible 19 points, for a possible total GET-D score of 26 points. The final GET-D, with ten criteria, is shown in the Figure.

To evaluate objective two (assessing raters’ perceptions of usability of the GET-D), the trained group of raters reviewed the scores assigned by all raters for each of the five training samples, and disagreements were discussed in detail. These discussions produced several clarifications to the training and GET-D instructions provided to potential raters. Additionally, we modified the GET-D instructions (shown in online Additional file 1: Appendix) to include more detail regarding each criteria and guidelines for scoring.

### Psychometric testing of the GET-D

After revising the instrument in its current format, we validated the GET-D with existing data from EPIC. We aimed to assess the inter-rater reliability and construct validity of the GET-D. The research team formed one hypothesis regarding the GET-D’s reliability and four additional hypotheses about construct validity. Criterion-based validity, or the comparison of the GET-D to a “gold standard”, was not assessed, because no standard measures of goal setting and action planning quality currently exist. However, two forms of construct validity could be assessed, including *nomological validity* (i.e., does the GET-D behave as expected with measures of variables that should have a relationship with goal-setting and action planning quality?) and *known groups validity* (i.e., can the GET-D produce relevant and expected group differences?) [21]. Our psychometric hypotheses and corresponding statistical expectations were as follows:

Hyp.1. The GET-D would demonstrate high inter-rater reliability between two raters for each of the ten criteria as evidenced by a Cohen’s Kappa greater than 0.70.

Hyp.2. Participants with greater patient activation scores at baseline and after the intervention would write higher quality goals and action plans (GET-D), as evidenced by a modest positive correlation between the measures.

Hyp.3. Based on previous research [7], we expected that participants with greater self-efficacy for diabetes self-management before and after the intervention period would write higher quality goals and action plans (GET-D), as evidenced by a strong positive correlation between the measures. We expected that the post-intervention self-efficacy scores would be more highly correlated with GET-D than those collected at baseline.

Hyp.4. Based on previous research [22], we expected that participants with lower self-reported depressive symptoms at baseline would write higher quality goals and action plans (GET-D) as evidenced by a modest negative correlation between the measures.

Hyp.5. Participants in the goal-setting intervention group (relative to comparison group) would have higher quality goals and action plans (GET-D), as evidenced by a comparison of group means.

We recruited and trained two physician-fellows to use the GET-D to rate each goal and action plan. Raw scores for each rater were used for calculations of inter-rater reliability using Cohen’s Kappa [23], except for the feasibility ratings, which were dichotomized (into low and high feasibility) for each rater prior to calculating inter-rater reliability. Averaged rater scores were calculated for each criterion and used in subsequent analyses.

Validation data for each study participant included three self-report measures collected as part of the study’s baseline measures. The Diabetes Self-Efficacy Scale (DSES) is an eight-item measure evaluating respondents’ confidence in performing specific diabetes management tasks, such as diet, exercise, blood glucose management, and lifestyle domains [24]. Individual DSES scores represent the mean value of all 8 items with higher scores corresponding to greater self-efficacy. The Patient Assessment of Chronic Illness Care (PACIC) measures respondents’ perception of how their health care providers’ communication and behavior are consistent with the elements of the Chronic Care Model [8,25]. One PACIC subscale is patient activation (meaning the health care provider was perceived as having encouraged greater patient involvement in their care and self-management). Individual PACIC patient activation scores represent the mean value of all subscale items, ranging from 1–5 with higher scores corresponding to greater encouragement. Both the DSES and the PACIC were collected again after the intervention period. The Depression Anxiety Stress Scale (DASS) is a 42-item self-report measure of anxiety, depression and stress with three distinct subscales [26]. Higher DASS scores indicated greater levels of depression and distress.

The patient activation subscale of PACIC, the full DSES, and the depression subscale of DASS were used in the testing of hypotheses 2–4, respectively. Pearson’s correlation coefficients were calculated for each of the construct validation measures with the overall GET-D and for each of the goal and action plan subscales of the GET-D. For hypothesis 5, we used descriptive statistics (t-test) to determine the statistical significance of the differences between the two study group means and standard deviations for the overall and subscale scores on the GET-D.

## Results

### Participants

Eighty-five EPIC participants completed the GET-D questionnaire. Overall, the 85 participants were between 63–84 years old, and less than half were white, non-Hispanic (See Table 1). 74.1% had more than a high school education. There were no significant differences between intervention or comparison participants.

**Table 1:** Characteristics of the study population (N=85)

**Characteristics**	**Goal-setting intervention arm (n=44)**	**Group education arm (n=41)**	**P-value**
Age, mean (SD)	64.6 (7.3)	63.6 (7.7)	0.57
Race, number (%)			
White	22 (50%)	17 (41%)	
African American	13 (30%)	14 (34%)	0.54
Hispanic or Latino	6 (13%)	9 (22%)	
Other	3 (7%)	1 (3%)	
Co-morbidity Score*, mean (SD)	2.98 (2.3)	3.66 (3.1)	0.25
Baseline Hemoglobin A_1_C, mean (SD)	8.53 (1.20)	8.65 (1.23)	0.67
Education Level, number (%)			
≤ High School	12 (27.3%)	10 (24.4%)	0.76
> Some College/Trade School	32 (72.7%)	31 (75.6%)	
Knowledge and Understanding of Diabetes^†^, mean (SD)	3.1 (.85)	2.9 (1.0)	0.54
How Much Diabetes Interferes with Daily Life^‡^, mean (SD)	2.5 (.77)	2.7 (.88)	0.36

*Deyo modification of the Charlson Co-morbidity Index using outpatient ICD-9 codes.

^†^Diabetes Care Profile^22^, section 4, (13-item subscale, scale range 1–5 [Higher score indicates greater knowledge]).

^‡^Diabetes Care Profile, section 7 (14-items subscale, scale range 1–5 [Higher score indicates more interference]).

### Psychometric analyses

The GET-D’s distribution of scores for each criterion is shown in Table 2. There were no missing data for any criterion. Some items (2b: Goal Presence of a Deadline, 4c: Action Plan Location, 4e: Action Plan Activity Monitoring) reflected clear “floor effects”, in which almost all raters’ scores were “0”, indicating an absence of the criterion in the participant’s goal or action plan. Overall, the distribution of each criterion was more restricted than desired reflecting the overall poor quality of the goals and action plans in our sample. Despite this, Hypothesis 1, that the GET-D would demonstrate high inter-rater reliability between two raters for each of the ten criteria as evidenced by a Cohen’s Kappa greater than 0.70, was largely supported. As shown in Table 2, nine of the ten criteria had Kappa values between 0.62 and 0.87, which are considered good to very good [27], with an average inter-rater reliability across all ten criteria of 0.66. The criteria Action Plan Location (4c) demonstrated a very low kappa of −0.01, reflecting the absence of any action plans in which both raters scored a “3”. However, raw agreement for this criterion, like all of the other criteria, was high (i.e., both raters scored 83 of 85 participants as a “0” on this criterion). Across the ten GET-D criteria, average raw agreement between the two raters was high (94.4%), ranging from 87% to 99%.

**Table 2:** Inter-rater scoring and agreement by item on the goal-evaluation tool

**Goal evaluation tool-diabetes (GET-D) items**	Frequency of scores*				**Raw agreement (%)**	**Kappa**
	**0**	**1**	**2**	**3**		
Goal 1: Relation to Self-Management	24	146			91%	0.62
Goal 2a: Measurable Specificity	128			42	95%	0.87
Goal 2b: Presence of a Deadline	164			6	98%	0.65
Action Plan 3: Relation to the Goal	55	115			87%	0.71
Action Plan 4a: Specific Action	54			116	91%	0.78
Action Plan 4b: Frequency or Schedule	145			25	96%	0.86
Action Plan 4c: Location	168			2	98%	−0.01
Action Plan 4d: Intensity or Duration	155			15	94%	0.63
Action Plan 4e: Activity Monitoring	167			3	99%	0.66
Action Plan 5: Feasibility	36	114	13	7	95%	0.77
***Average across all 10 Criteria***					***94.4%***	***0.66***

Legend:

*Criteria 1–4 were scored as either 0,1 or 0,3, to weight some criteria as more critical than others. Criteria 5 was scored as 0,1,2,3. The distribution of scores is based on scores provided by both raters (n=170 total, n=85 for each rater). There were no missing data for any criteria.

Hypotheses 2–4 tested the construct validity of the GET-D. We expected that participants with higher quality goals and action plans (GET-D) would also have higher patient activation scores (PACIC), greater self-efficacy for diabetes self-management (DSES), and lower reported depression (DASS). As shown in Table 3, these hypotheses were partially supported. Though goal setting was not significantly correlated to any measure, action plans and GET-D total scores were significantly related to the validation measures as expected, though the values of the correlations were attenuated by the lower quality goals and action plans (i.e. floor effects) in our sample. Finally, in Hypothesis 5, we assessed known-groups validity by comparing the quality of goal-setting and action planning among the participants in the intervention group to those in the comparison group. As shown in lower portion of Table 3, the intervention group had significantly (p<.01) higher total GET-D scores than the comparison group, driven primarily by differences in action plan quality.

**Table 3:** Validation Results for GET-D with 85 participants

	**GET-D Goal Sub-Total**	**GET-D Action Plan Sub-Total**	**GET-D Total**
***Correlation***^***^***^***with***			
**Patient Activation (PACIC) at Baseline** (n=85)	.078	.331**	.252*
**Patient Activation (PACIC) after intervention** (n=84)	-.063	.306**	.200
**Self Efficacy (DSES) at Baseline** (n=85)	.176	.262**	.222*
**Self Efficacy (DSES) after intervention** (n=84)	.192	.385***	.376***
**Depression (DASS) at Baseline** (n=85)	-.190	-.358**	-.325**
**Mean (SD)*by Treatment Group***^***&***^			
**Intervention Group** (n=44)	1.98 (1.6)	5.35 (3.4)*	7.33 (4.4)**
**Comparison Group** (n=41)	1.41 (1.4)	3.51 (3.2)	4.93 (3.9)

Legend: * <.05, ** <.01, *** <.001.

^^^ Pearson Correlation, ^&^ T-test.

## Discussion

The absence of tools to reliably assess the quality of patients’ reported goals and action plans creates a critical methodological gap for self-management research, and ultimately, for chronic care outcomes. In this study, we describe a tool that begins to fill this gap. The GET-D demonstrates both reliability between raters and validity in assessing rater-determined quality measured against theoretical goal-setting criteria. This suggests that trained raters could consistently and adequately judge goal-setting and action planning quality based on nothing more than patients’ written goals and action plans. Further, the statistical relationships between the GET-D and our validation measures reflect the theoretical relationships we expected. Self-efficacy had the strongest positive relationship with the GET-D ratings, particularly after the intervention. Self-efficacy has often been described as a mediator between setting and achieving goals [7] and our data partially support this [19]. The decrease in the relationship between GET-D and patient activation from baseline to post-intervention was initially surprising, but upon reflection, is intriguing. It suggests that those participants with more encouraging providers at baseline (as compared to post-intervention) were more likely to produce higher GET-D scores after the intervention, emphasizing the importance of collaborative, supportive care as foundational to later outcomes. The negative relationship between depression and GET-D ratings was much stronger than expected, but consistent with prior goal-setting studies [9], and the literature describing the association of diabetes, depression, and diabetes care related-distress [22]. Finally, the GET-D could distinguish between participants who received the EPIC goal-setting intervention and those who received a traditional diabetes education intervention [19]. These patterns suggest that the GET-D is measuring constructs we intended it to measure.

Our assessment of the validity of the GET-D is not without limitations. The EPIC participants’ (who were older and relatively educated) goals and action plans were uniformly of lower quality than expected, creating floor effects in the data. This was particularly evident in the action-planning criterion “location”, regarding where proposed activities would take place; few action plans included this information. As such, we elected to retain this criterion in the GET-D, pending additional validation studies. Though most criteria did not reflect the severe floor effects of this specific criterion, the data overall reflected lower quality for goal setting and action plans than desirable for optimal validation, attenuating the statistical relationships we measured. These floor effects also inhibited our ability to discern the relative contribution of goal versus action plan criteria in our validation analyses, and as such we focused on the overall GET-D score. The floor effects likely arise from the structure of the GET-D questionnaire because patients are not guided by the scoring criteria when developing their goals and action plans. This design element was deliberate, and reflects the psychological literature on motivation [7,9,10]. The clinical relevance of this contention is that artificially improving GET-D scores by providing patients with a scoring “cheat-sheet” may cause the GET-D to lose its predictive and construct validity. While the GET-D should be further assessed on a higher quality sample (and perhaps reflect a different population of goal-setters), the instrument performed well in spite of our data sample. The GET-D serves as a useful first step for a rigorous evaluation of the potential effectiveness of goal-setting in diabetes self-management. Additional studies with larger and more diverse patient samples are needed to further validate the GET-D. Subsequent studies should evaluate the relationship between goal-setting quality and the likelihood of goal-attainment and improvement in clinical diabetes outcomes.

Like most observer rating scales, the GET-D requires some training to use properly and promote consistency across raters. We developed a relatively brief training session that can be easily used by other researchers and educators. Second, we observed common rater perspectives with which trainers should be familiar, namely, that of the severe rater who takes a very literal approach to using the GET-D and that of the lenient rater who wants to give the participant the benefit of the doubt. Despite this finding, raters of both types were easily trainable through our brief session. Though we utilized physician-fellows as our final raters (due to their availability), the scale was tested on a variety of raters in its piloting phase (including lay persons and clinicians) and does not require advanced medical knowledge for use.

## Conclusions

In conclusion, goal-setting is frequently proposed as the mechanism to enhance diabetes self-management, but evidence to suggest that goal-setting is beneficial is mixed [7,19]. Measures for evaluating the quality of goals and action plans, the principal mediator of goal-setting effectiveness, are limited [5]. The GET-D is a reliable and valid tool for rating the quality of patients’ written goals and action plans and can potentially address this critical gap.

### Implications

The GET-D holds promise as a measure of intervention fidelity for a range of clinical interventions that seek to raise the performance of self-management behaviors and improve clinical outcomes. The GET-D may also be a useful tool for measuring self-management in routine care as part of a comprehensive approach to diabetes care. Diabetes educators, clinicians, and a variety of other providers could be trained in the scoring and monitoring of diabetes goal-setting using the GET-D. Future studies should test the use of the GET-D in this context. Finally, the GET-D may serve as a template for assessing the quality of goal-setting and action planning among other chronic conditions, such as asthma and heart failure. Further work is needed to better measure and draw connections between goal setting interventions and outcomes in the setting of chronic illness.

## Competing interests

The authors declare that they have no competing interests relating to the GET-D or this manuscript.

## Authors’ contributions

CT: conception and design of GET-D, data analysis, drafting and critical revisions to the article. AD: funding for original study, conception and design of GET-D, critical revisions to the article. PH: conception and design of GET-D, critical revisions to the article. AB: data analysis, critical revisions to the article. ER: data collection, data analysis, critical revisions to the article. All authors read and approved the final manuscript.

## Pre-publication history

The pre-publication history for this paper can be accessed here:

http://www.biomedcentral.com/1472-6947/12/152/prepub

## Supplementary Material

Additional file 1 **Appendix.** Goal Evaluation Tool for Diabetes (GET-D) *A Rubric for Evaluating the Quality of Participant Goal Setting.* Click here for file
