# Supplementary material for: Measuring the quality of Patients’ goals and action plans: development and validation of a novel tool
Source: BMC Med Inform Decis Mak. 2012 Dec 27;12:152. doi: 10.1186/1472-6947-12-152 (PMC3544573; doi:10.1186/1472-6947-12-152)

Scored by □□□ Date ___/____/____ Patient ID □□□□

Appendix: Goal Evaluation Tool for Diabetes (GET-D)

A Rubric for Evaluating the Quality of Participant Goal Setting

(*Questionnaire given to participants*)

The following questions, allow us to know about your goal for your diabetes care and what you plan to do to reach this goal.

1) Beings as specific and detailed as possible please describe your diabetes goal.

____

____

____

____

____

____

____

____

____

2) Being as specific and detailed as possible, what actions will you take to reach your diabetes goal.

____

____

____

____

____

____

____

GET-D: Rater Scoring Sheet, Definitions and Rater Instructions

NOTE: If a patient describes more than one goal, evaluate the elements of #2 below for each goal and score ONLY the best scoring goal.

ASSESSING THE GOAL: *Scoring*

1. **Is the GOAL a diabetes-related self-management task?**[i]

   IF YES (*e.g., to manage diabetes better, to lose weight, to take meds regularly*), Score = 1

   IF NO, or in the absence of any goal, Score = 0

   Participant Score for #1 (total of 1 pts is possible) [ ]

2. **Does the GOAL include elements taught in the intervention?**

   a. Does the goal identify a specific observable/measurable outcome?[ii]

   (*e.g., to lower A1C by 1 pt, lose 10 pounds, lower cholesterol by 20 points*)Yes = 3; No = 0 [ ]

   b. Does the goal identify a specific time frame for achieving the goal?

   (i.e., a deadline; *e.g., in 3 months, within 1 week, by September 2009)* Yes = 3; No = 0 [ ]

   Participant Score for #2 (total of 6 pts is possible) [ ]

Participant Score for ASSESSING THE GOAL (total of 7 pts is possible) [ ]

NOTE: If a patient describes more than one action plan, evaluate the elements of #4 below for each action plan and score ONLY the best scoring action plan. The best scoring action plan may not be an activity that helps reach the goal scored above (and it would score a 0 on #3), but that is preferable to choosing a goal that matches but demonstrates poorer grasp of the 5 elements below.

ASSESSING THE ACTION PLAN:

3. **Is the ACTION PLAN a** [iii]**:**

   Plan with activities that, if followed, could be used to reach the stated goal? If Yes = 1

   Restatement of goal without new activities OR not consistent with the goal? If Yes = 0

   Participant Score for #4 (total of 1 pt is possible) [ ]

4. **Does the ACTION PLAN include elements taught in the intervention?**

   a. Does the plan identify specific actions or activities that could help reach the goal?[iv]

   (*e.g., exercise, walk, take meds, eat more or less of certain foods)* Yes = 3; or No = 0 [ ]

   b. Does the plan identify how often the action(s) will be taken to reach goal?

   (*This is schedule or frequency, e.g., daily, weekly, at 5 pm, in the morning)* Yes = 3; No = 0 [ ]

   c. Does the plan identify where the action(s) will occur?

   (*e.g., at the park, while at home, in kitchen, at the yoga studio*) Yes = 3; No = 0 [ ]

   d. Does the plan identify with what intensity the action(s) will occur?

   (*This might be duration, e.g., 30 mins, with XX heart rate, until calm*) Yes = 3; No = 0 [ ]

   e. Does the plan identify how the participant will monitor (i.e., keep track of) if the action(s) have taken place OR if the goal has been reached?

   (*e.g., keep a log, weigh, take heart rate, track distance walked*) Yes = 3; No = 0 [ ]

   Participant Score for #5 (total of 15 pts is possible) [ ]

5. **Does carrying out the overall ACTION PLAN (5a-5d) appear feasible**[v]**?**

   SEE decision tree.

   Participant Score for #6 (total of 3 pts is possible) [ ]

Participant Score for ASSESSING the ACTION PLAN (total of 19 pts is possible) [ ]

Total for ASSESSING THE GOAL AND THE ACTION PLAN (26 pts possible) [ ]

Scored by □□□ Date ___/____/____ Patient ID □□□□

[i] The GOAL may address a direct diabetes self-management task (as defined by providers) such as weight loss, medication management, etc. However, it may also address a goal of a behavior or task that indirectly affects ability to manage diabetes (as defined by patient/participant). An example list of potential self-management tasks that are acceptable to the researchers is provided immediately below. <u>However, this list may not be all inclusive and other answers may be appropriate.</u> EXAMPLES: Monitor important symptoms, use medications correctly, manage medical emergencies, eat healthy diet, lose weight, exercise or stay physically active, reduce stress, talk to and question physician, identify or use resources in community, meet responsibilities at work or home, adjust to physical limits, get support from family or friends, manage emotional reactions, identify or use hospital resources, handle finances or benefits, manage pain, improve sleep, develop hobbies or leisure activities, improve sexual relationship, take care of spiritual or religious needs, stop smoking, reduce alcohol intake, etc.

[ii] A GOAL'S observable outcome should be measurable, and in most cases will include numeric information. Specific numeric information about outcome goals (e.g., reach 215 lbs, lose 15 lbs) is acceptable even if we don't know the beginning reference point. Outcomes may be identified that are not measureable or observable might include more generic or vague statements (e.g., lose "some" weight, eat "less" sugar).

[iii] ACTION PLANS must include new or additional (more than what is in the goal) activities or actions that, if followed, could lead to the stated goal. This is no matter how specific or detailed the action plan is; these aspects are assessed in #5. **SCORING NOTE**: If so, the action plan will seem logical or "consistent with stated goal" (though potentially incomplete) and scores a "1." If not, the action plan: a) may include no new actions or activities, and merely restate the goal, without answering "how?" the goal will be reached; or b) may include actions or activities that are "inconsistent" with the stated goal and carrying out the activities (*e.g., drinking 8 glasses of water*) would not help reach the stated goal (*e.g. sleeping more regularly*). Either of these would score a "0." If multiple actions are provided, and some do not correspond to the stated goal, score those that do correspond.

[iv] ACTIONS do not have to be written as "steps" – and participants should not score higher if they are. Some action plans will be more "step-wise" and include more detail, but the reviewer should focus on whether the action plan includes specific actions or activities that could help reach the goal, not the degree to which these are logically ordered.

[v] <u>Feasible vs. Unfeasible</u>: *Feasibility cannot be judged until the elements of the action plan have been scored.* The essential question: Can the GOAL be reached if put into practice just as written? If so, it is a more *feasible* goal; if not is a more *unfeasible* goal. It is essential that the reviewer score based on <u>absolute achievability</u> in reaching the measurable outcome within the specified time frame (if available) or at all (e.g, can this goal be reached?). The reviewer should not score on <u>contextual achievability</u> (e.g., can this particular person, with their available resources and commitments, reach this goal?). This score requires some reviewer judgment but more importantly, consistency in following the **<u>decision tree</u> below** to determine feasibility. Note that this overall feasibility assessment should not include monitoring – only activities, frequency, intensity, and location. Monitoring is not included because the plan could be effective and feasible without monitoring.

<u>Feasibility Decision Tree:</u>

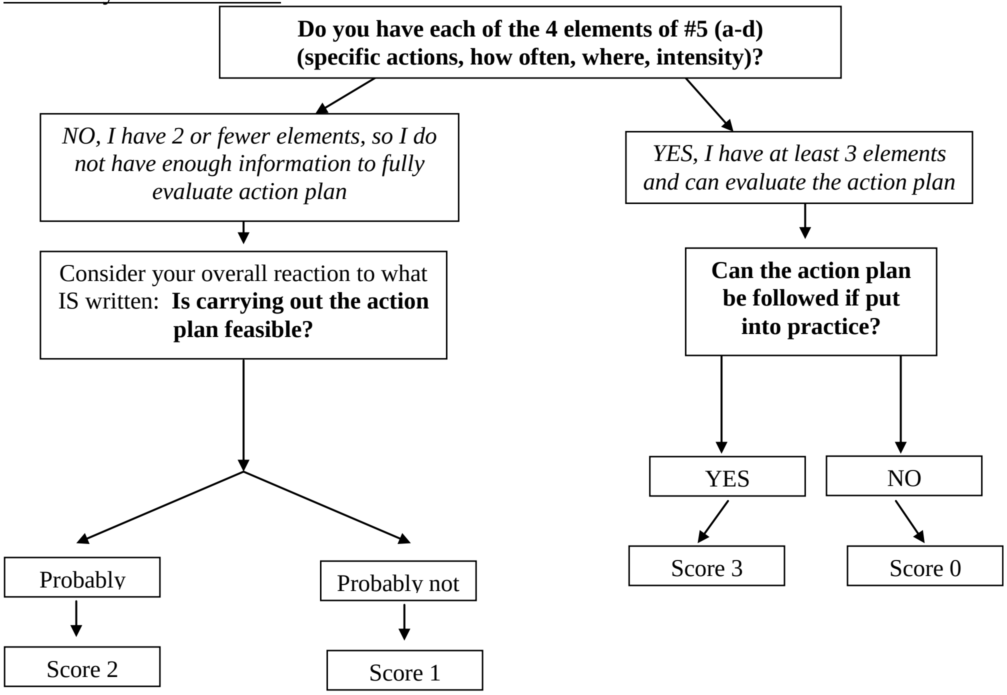

Supplement: Additional file 1 — Appendix. Goal Evaluation Tool for Diabetes (GET-D) A Rubric for Evaluating the Quality of Participant Goal Setting. [file 1472-6947-12-152-S1.pdf]
